# Continuous non‐contact respiratory rate and tidal volume monitoring using a Depth Sensing Camera

**DOI:** 10.1007/s10877-021-00691-3

**Published:** 2021-03-20

**Authors:** Paul S. Addison, Philip Smit, Dominique Jacquel, Anthony P. Addison, Cyndy Miller, Gardner Kimm

**Affiliations:** 1 Video Biosignals Group, Patient Monitoring, Medtronic, Technopole Centre, Edinburgh, EH26 0PJ UK; 2 Respiratory Interventions, Medtronic, Ventilation, Carlsbad, CA USA

**Keywords:** Respiratory rate, Tidal volume, Respiratory function, COVID-19, Depth sensing camera

## Abstract

The monitoring of respiratory parameters is important across many areas of care within the hospital. Here we report on the performance of a depth-sensing camera system for the continuous non-contact monitoring of Respiratory Rate (RR) and Tidal Volume (TV), where these parameters were compared to a ventilator reference. Depth sensing data streams were acquired and processed over a series of runs on a single volunteer comprising a range of respiratory rates and tidal volumes to generate depth-based respiratory rate (RR_depth_) and tidal volume (TV_depth_) estimates. The bias and root mean squared difference (RMSD) accuracy between RR_depth_ and the ventilator reference, RR_vent_, across the whole data set was found to be -0.02 breaths/min and 0.51 breaths/min respectively. The least squares fit regression equation was determined to be: RR_depth_ = 0.96 × RR_vent_ + 0.57 breaths/min and the resulting Pearson correlation coefficient, R, was 0.98 (p < 0.001). Correspondingly, the bias and root mean squared difference (RMSD) accuracy between TV_depth_ and the reference TV_vent_ across the whole data set was found to be − 0.21 L and 0.23 L respectively. The least squares fit regression equation was determined to be: TV_depth_ = 0.79 × TV_vent_—0.01 L and the resulting Pearson correlation coefficient, R, was 0.92 (p < 0.001). In conclusion, a high degree of agreement was found between the depth-based respiration rate and its ventilator reference, indicating that RR_depth_ is a promising modality for the accurate non-contact respiratory rate monitoring in the clinical setting. In addition, a high degree of correlation between depth-based tidal volume and its ventilator reference was found, indicating that TV_depth_ may provide a useful monitor of tidal volume trending in practice. Future work should aim to further test these parameters in the clinical setting.

## Introduction

The measurement of respiratory function is important in the hospital setting as it relates to numerous disease states and may be indicative of ensuing issues. Changes in Respiratory Rate (RR) may correlate with major complications such as respiratory tract infections, respiratory depression associated with opioid consumption, anaesthesia and/or sedation, as well as respiratory failure [1–3]. In addition, many early warning scores (EWS), MEWS, NEWS, etc., incorporate respiratory rate (RR) within the scoring system [4]. Tidal Volume (TV) is less often measured in practice, as it requires a sealed mask or intubation for measurement purposes. However, along with its counterparts of RR, SpO_2_ and PaCO_2_, it is recognized as a critical parameter in understanding pathophysiologic patterns of death which evolve due to sepsis, congestive heart failure, pulmonary embolism, hypoventilation, narcotic overdose, and sleep apnea [5]. Depth cameras are emerging as a tool that can provide a continuous measure of both respiratory rate and tidal volume. They do so by first deriving a respiratory volume (RV) signal from the respiratory motions of the patient from which these parameters can be extracted. The non-contact monitoring of RR and TV would prove valuable in the monitoring of viral pandemics, including novel coronavirus (COVID-19) patients, as well as those with other viral respiratory tract diseases, where minimum contact with the patient is desired and a robust measurement is essential [6, 7].

Many studies have focused on the measurement of respiratory rate from the depth camera. Martinez & Stiefelhagen [8] assessed 67 healthy patients in a sleep lab where the subjects were allowed to use at will: blankets of various thicknesses, various sizes and amounts of pillows, books, newspaper and magazines, etc. during the recording. They found that they could determine RR within 1 breath/min 88.7% of the time. Another sleep study by Yu et al. [9, 10] concerned a participant monitored over 10 nights, resulting in a total of 42 h of sleep data. The subject slept with a blanket for 5 days, and 5 days without a blanket. They achieved a 92% accuracy in their measurements. Our own group measured continuous RR during an acute hypoxic challenge during an oximeter breathe-down study [11]. The hypoxic challenge elicited a wide range of respiratory rates and patterns in the respiratory volume signal. A bias and RMSD of 0.04 and 0.66 breaths/min respectively were found against a capnograph reference RR signal. In addition, a high correlation (R = − 0.99) was found between two RR measurements. Seppanen et al. [12] measured the respiratory rate of eight volunteers who were instructed to follow a variety of breathing patterns while being monitored. They used regions of interest (ROIs) which mimicked chest/abdomen bands used in sleep studies. They found small absolute errors of between 0.26 and 0.30% when comparing their measurement of RR_depth_ to that of a spirometer. Bernacchia et al. [13] found good agreement between the breath periods derived from a Kinect depth sensing system and a spirometer reference in a study of 10 healthy young adult subjects asked to maintain ‘regular respiratory activity’. An RMSD of 9.7% RMSD was obtained for the breath periods between the two devices. Centonze et al. [14] used a Kinect system to continuously monitor a single patient over 8 h. An average error in frequency was calculated to be 0.87% when compared to a polysomnographic record. Al-Naji et al. [15] found excellent agreement between depth-sensing RR and a piezo-belt reference in a study of five children (ages 1 to 5 years). Bland–Altman analysis revealed limits of agreement ranging from [− 0.91 to + 1.0] to [− 1.3 to 2.3] breaths/min for scenarios with and without blankets respectively. High degrees of correlation (ranging from 0.97 to 0.99) were also found, where the small variation in correlation was due to background lighting levels and whether bed sheets were used. Cenci et al. [16] studied breath period (rather than RR) in three preterm infants, assessed in five 30 s intervals. They found excellent agreement (R = 0.95) with a reference derived from ECG impedance pneumography. Two children in the Pediatric Intensive Care Unit (PICU) (4 months and 1 year old) were studied by Rehouma et al. [17]. The patients were ventilated, and the ventilator RR was used as the reference over five 1-min data acquisition periods. They reported RMSDs of 0.77 and 0.68 breaths/min for the two patients.

Although many studies have focused on the RV signal and its correlation with the respiratory waveform from a reference device, the measurement of tidal volume (peak-to-trough breath measurement in spontaneous breathing) from the RV signal is less common in the literature. Aoki et al. [18] studied 4 healthy volunteers who were instructed to vary their respiratory flow over 180 s measurement epochs while being monitored in a sitting position by a Kinect depth camera. Correlation coefficients of 0.99 were obtained for subjects for the measured TV relative to a flow reference obtained using an expiration gas analyser. (Per-subject scatter plots and corresponding Bland–Altman plots with limits of agreement drawn on were provided in the paper, although no statistical measures of error such as mean bias, RMSD, or limits of agreement were stated by the authors.) Oh et al. [19] studied 10 healthy adult volunteers comparing the depth tidal volume against a ventilator reference. They obtained a correlation coefficient and mean tidal volume error of 0.98 and 8.1% respectively. This was achieved by combining both spatial and temporal information within their method. In addition to RR, Rehouma et al. [17] measured the tidal volume of two neonatal patients requiring ventilator support for breathing in the PICU using a Kinect™ V2 system. They obtained mean RMSDs of 5.4 and 6.4 ml between their depth-based method and the ventilator reference for the neonates aged 4 months and 1 year respectively. Other clinically relevant respiratory volume measures were derived by Soleimani et al. [20] in 40 Chronic Obstructive Pumonary Disease (COPD) patients using a Kinect™ V2 camera. For each patient at least 3 forced vital capacities (FVCs) and 3 slow vital capacities (SVCs) were recorded and compared to spirometer measurements. Correlation coefficients of 0.999 were found for both SVC and FVC. The mean/standard deviation of the differences was calculated to be 0.029 / 0.049 and 0.009 / 0.039 L for SVC and FVC respectively. In another investigation by the same group, Sharp et al. [21], studied 100 patients from a general respiratory physiology laboratory with a variety of lung issues. They found that their method tracked estimated forced vital capacity (FVC) and vital capacity to within ± 1% but forced expiration volume (FEV) did not demonstrate acceptable limits of agreement, with 61.9% of readings showing more than 150 ml difference.

The work reported here extends current research in this area through a study of both respiratory rate and tidal volume of a subject over a range of normal breathing activity on a single subject. Performance metrics associated with both accuracy and trending behaviours are assessed against high-quality reference RR and TVs obtained using a ventilator flowmeter.

## Methods

### Data acquisition and processing

The data was collected over a series of ten separate runs where the volunteer breathed on a ventilator (Puritan Bennet™ 980, Medtronic, Carlsbad, CA). This provided a highly accurate refence for respiratory rate and tidal volume. The subject was connected to the ventilator using a standard adult breathing circuit (Medtronic/DAR part number 301/6326), mechanical filter (Medtronic/DAR part number 351U5856), and a mouthpiece. The ventilator mode was set to SPONT (spontaneous ventilation) with a pressure support level of 5 cmH_2_O, PEEP (positive end expiratory pressure) of 5 cmH2O, and oxygen concentration setting of 21%. The inspiratory trigger setting for the ventilator was set at a level to prevent false triggers. Respiratory rate and tidal volume are measured by the ventilator using flow sensors in both the inspiratory and expiratory gas pathways internal to the ventilator and are displayed under BTPS (body temperature and pressure saturated) conditions. A video camera was used to capture the ventilator measured values for respiratory rate and tidal volume from the ventilator display screen for later comparison. The volunteer undertook spontaneous breathing comprising respiratory rates in the range between 10 and 20 breaths/min and tidal volumes comprising a range from shallow to deep breaths. It should be noted that the data presented here was acquired prior to the current COVID 19 pandemic and our intention was to acquire data from additional volunteers. However, due to significant restrictions imposed on our work due to the pandemic, we were not able to perform this experiment on more volunteers. In light of this, and after internal discussion concerning the relevance and importance of the results, we took the decision to make the results publicly available. Testing was therefore conducted using a single normal healthy subject. The subject was 58 years of age, weighed 75 kg, and had no previous history of tobacco usage or other medical conditions that would affect respiratory lung mechanics. During the test runs the subject was instructed to purposely vary his breathing pattern in order to simulate a range of tidal volumes. Ten runs were conducted over a range of periods, from 45 to 270 s in length, where each run period was dictated by the tolerance of the volunteer to the respiratory activity. Depth data was captured using a Kinect V2 camera (Microsoft Corporation, Redmond, WA, USA) connected to a laptop and at a frame rate of 30 fps. The camera was mounted on a tripod and placed at approximately 1.5 m above the subject who laid in a supine position. The room was illuminated with standard ceiling mounted fluorescent lights. Other than starting and stopping the recording process, no other intervention or calibration was required over the study period.

**Fig. 1:**
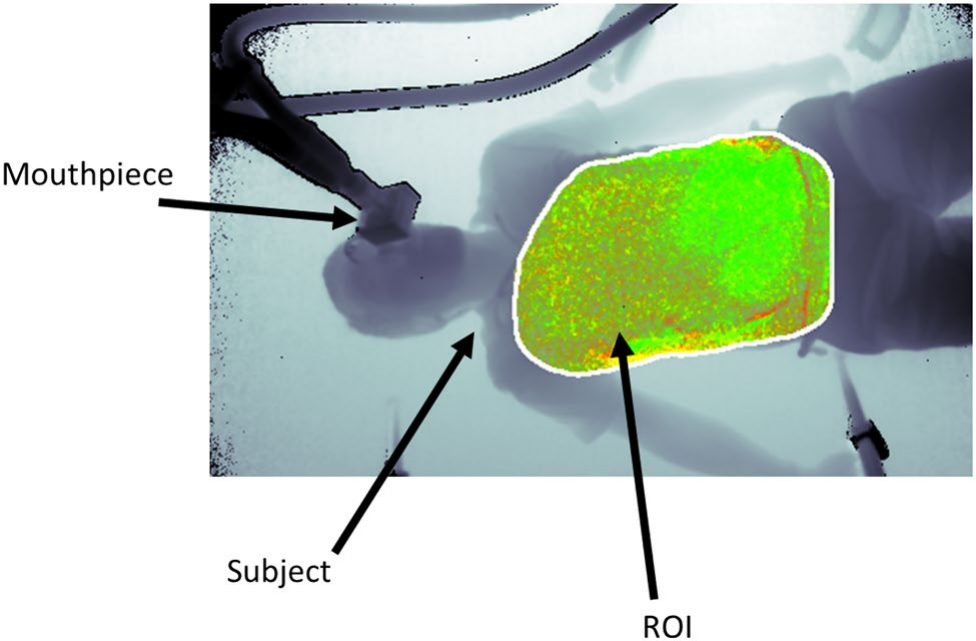
Depth image showing the subject with mouthpiece and ROI

**Fig. 2:**
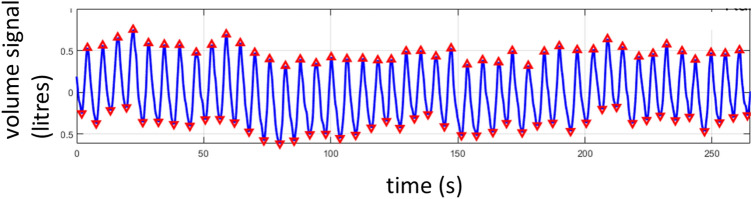
Respiratory volume signal from a typical run showing respiratory modulations with detected peaks and troughs indicated

**Fig. 3:**
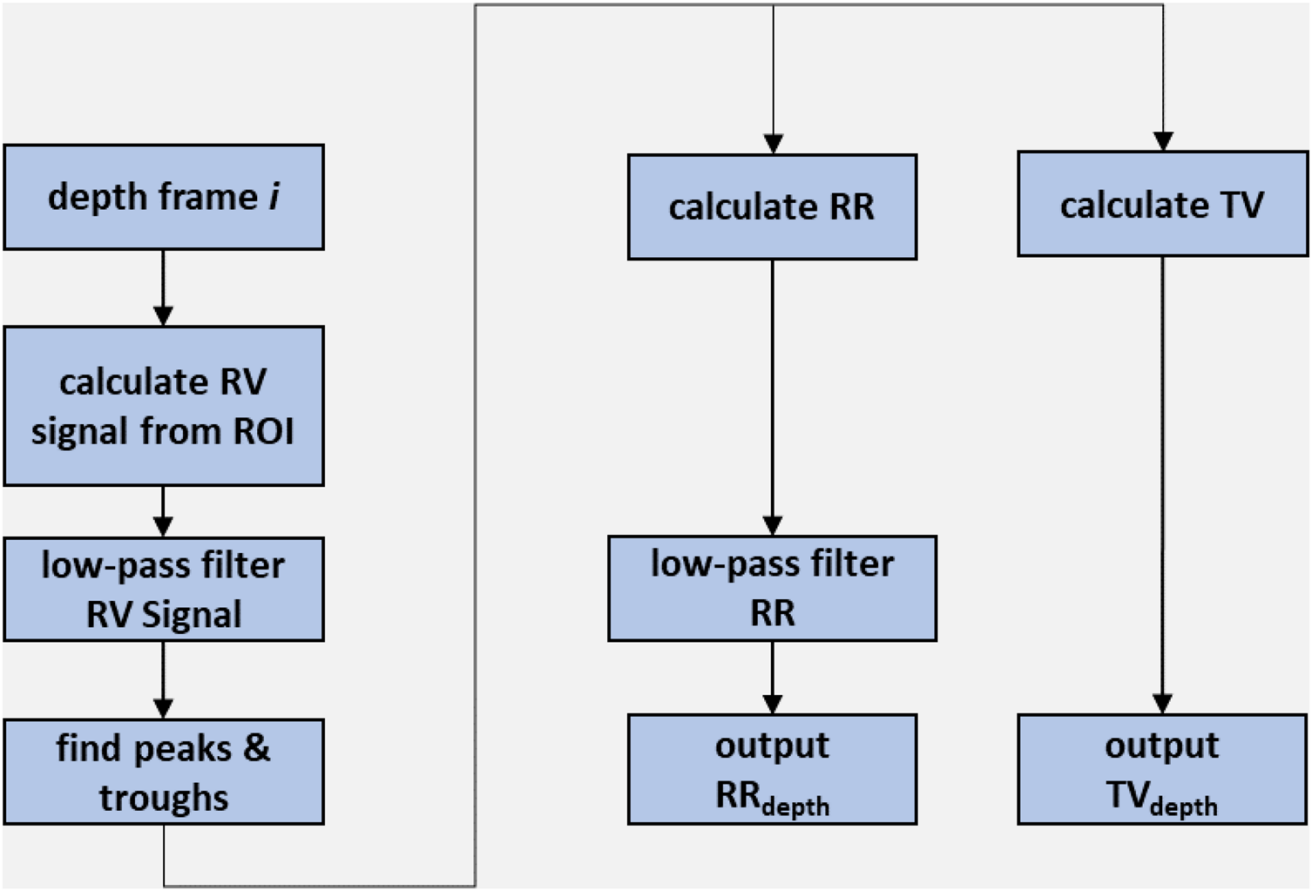
Algorithm flow diagram

A depth camera measures the distance to the surface of all objects within its field of view (FOV) and outputs a single matrix of distances (or depths) for each image frame. A respiratory volume (RV) signal was generated from the depth data image sequences by integrating the change in depths across a region of interest defined on the subject’s chest region over time (i.e. frame-by-frame). Figure 1 shows the subject in the depth image with the ROI shown defined on the torso during one of the experiments. ROIs may be generated in several ways. The simplest is to use the whole depth image. Alternatively, the user can define a box around the chest region. More sophisticated methods may be used to automate the ROI placement. The ROI generated in Fig. 1 uses a flood fill method. The RV signal was generated by integrating changes in depths across the area defined within the ROI over time. Figure 2 shows an example of one of the RV signals generated during the study where the identified peaks and troughs are indicated. These were determined using a peak detection algorithm. We calculated respiratory rate using the peak-to-peak breath periods extracted from the RV signal and a tidal volume (TV) from the peak-to-trough changes in volume extracted from the RV signal. The algorithm for calculating RR and TV is shown schematically in the flow diagram of Fig. 3. Reference signals for respiratory rate and tidal volume were obtained from the ventilator. These were synchronized with the output from the depth-sensing algorithm and used in the performance analysis. Note that data was analysed when both RR and TV were available as RR took slightly longer to begin reporting due to its additional low pass filter smoothing (usually a few seconds later than tidal volume which was computed breath-to-breath). This additional low pass filter is shown in Fig. 3. In this way we matched our results temporally.

### Data Analysis

*Bias* and *accuracy* statistics were calculated to compare the depth data derived RR and TV with that of the reference (ventilator) system. These are, respectively, the mean difference (bias) and the root mean squared difference (RMSD) between the test and reference values. That is (using RR as an example):

1 $$bias=\frac{\sum_{i=1}^{N}\left({RR}_{depth}(i)-{RR}_{vent}(i)\right)}{N}$$

and

2 $${RMSD \, accuracy = \sqrt{\frac{\sum_{i=1}^{N}{\left({RR}_{depth}(i)-R{R}_{vent}(i)\right)}^{2}}{N}}}$$

The RMSD represents a combination of the systematic and random components of the differences between the corresponding readings from the two devices. Least-squares linear regression was performed to obtain the line of best fit between the video and reference parameters from which the gradient, intercept, and Pearson correlation coefficient, R, was computed. A Bland–Altman analysis of the data was also performed using the method of Bland and Altman [22]. The corresponding limits of agreement were calculated using this methodology. Distributions of breathing rates are represented as normalized counts which sum to unity, in effect providing a discrete probability distribution of rates. The analysis of trending behaviour was carried out using a concordance plot where the difference between the two parameters, e.g. Δx and Δy, over a running time window are plotted against each other. A concordance value is then computed by calculating the percentage of points lying in the quadrants where both parameters have the same sign, (i.e. both positive or both negative), which indicates co-trending behaviour [24]. Matlab (R2018b) was used to process the data and perform the statistical analysis. An in-house developed C +  + application was used to capture the depth data.

## Results

**Fig. 4:**
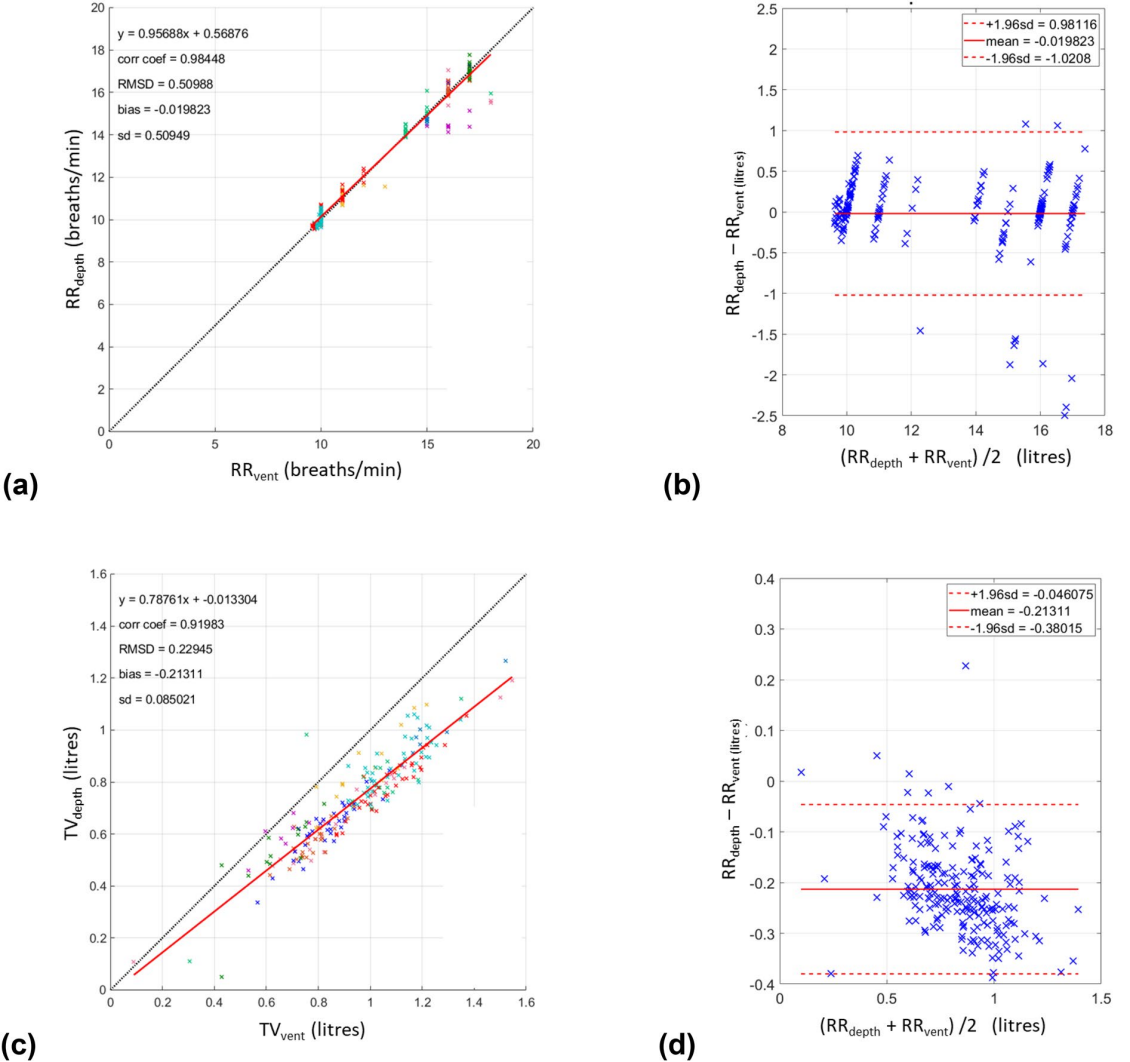
Scatterplots and Bland Altman plots of respiratory rates and tidal volumes. The colours of the data points in the scatter plots indicate separate runs

Figure 4a contains the plot of RR_depth_ against RR_vent_ pooled together for all ten experimental runs. A Pearson correlation coefficient of 0.98 (p < 0.001) was achieved and a line of best fit given by RR_depth_ = 0.96 × RR_vent_ + 0.57 breaths/min. The overall RMSD across the runs was 0.51 breaths/min with a corresponding bias of -0.02 breaths/min respectively. The associated Bland–Altman plot, illustrated in Fig. 4b, shows limits of agreement of − 1.02 to 0.98 breaths/min. The near zero mean bias is also obvious in this plot.

Figure 4c contains the plot of TV_depth_ against TV_vent_ for the same runs as Fig. 4a. A Pearson correlation coefficient of 0.92 (p < 0.001) was achieved together with a line of best fit given by TV_depth_ = 0.79 × TV_vent_—0.01 L. The overall RMSD across the runs was 0.23 L with a corresponding bias of -0.21 L. The associated Bland–Altman plot, illustrated in Fig. 4d, shows limits of agreement of − 0.38 to − 0.05 L. This time a distinct bias of the data is obvious in the plot.

**Fig. 5:**
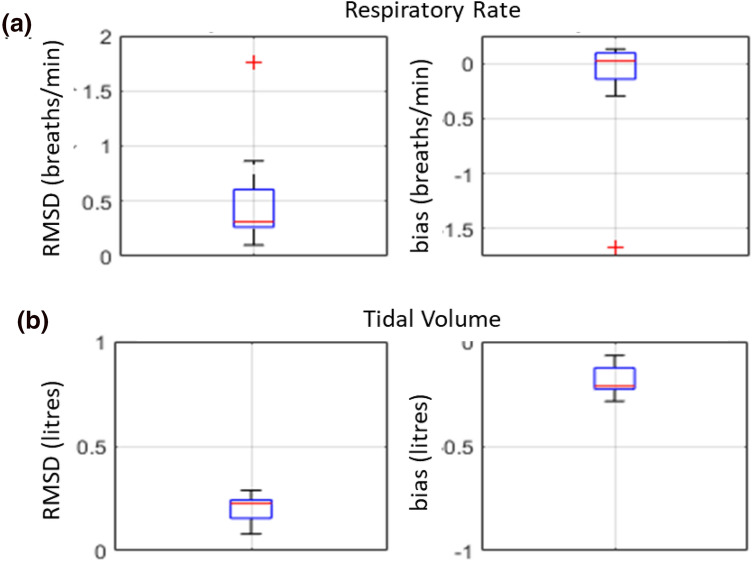
Boxplots of distributions of RMSD and bias across all 10 runs for **a** respiratory rate and **b** tidal volume

The spread of the RMSD and bias accuracy statistics on a per run basis for the ten experimental runs are presented as box plots in Fig. 5. The box plots of Fig. 5a show the spread of the individual mean biases and RMSDs for respiratory rate. These range from -1.67 to 0.13 (median = 0.22) and 0.10 to 1.76 (median = 0.31) breaths/min respectively. The box plots of Fig. 5b show the spread of the individual mean biases and RMSDs for the tidal volume for the ten experimental runs. These range from − 0.28 to − 0.06 (median = − 0.21) and 0.08 to 0.28 (median = 0.23) litres respectively.

**Fig. 6:**
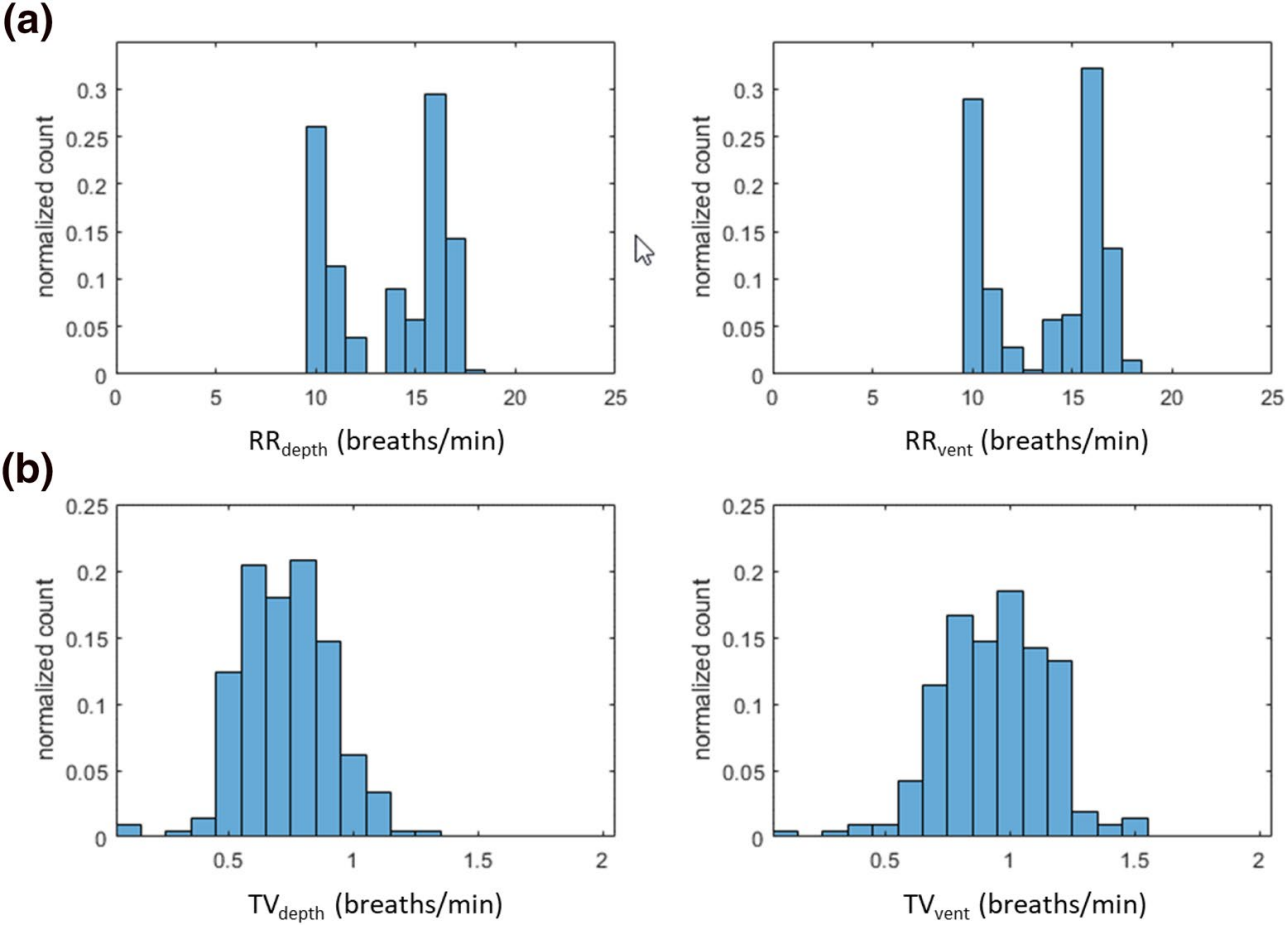
Distribution plots of **a** respiratory rates and **b** tidal volumes. Left-hand plots: depth camera results. Right-hand plots: reference values

The distributions of the individual respiratory rates and tidal volumes across all subjects for the depth sensing and reference ventilator devices are shown in Fig. 6a and b respectively. It can be observed that the distributions for each parameter exhibit similar morphologies. The respiratory rates of Fig. 6a exhibit near identical distributions. However, a shift in the tidal volume data to lower values for the depth-based measurement relative to the reference can be observed in Fig. 6b.

## Discussion

The study demonstrated a high degree of agreement between the respiratory rate determined from the depth camera and the reference signal from a ventilator under test conditions, where an RMSD of 0.51 breaths/min was achieved which corresponded to tight limits of agreement of − 1.02 to 0.98 breaths/min. This was consistent across each of the individual experimental runs. These results are very similar in value to others who have reported RMSD or limits of agreement, including those of Al-Naji et al. [15], Rehouma et al. [17], and an earlier experiment we conducted with healthy volunteers in a breathe-down study used in pulse oximetry [11]. Interestingly, a recent study by Breteler et al. [23] compared the respiratory rate from three wearable technologies and a mattress sensor in the clinical environment. They reported Bland–Altman limits of agreements of − 5.6 to 6.4 breaths/min for the mattress sensor and − 6.6 to 6.3 breaths/min for the wearable sensor with the tightest limits of agreement, with most of the respiratory rates falling in the range of 10 to 20 breaths/min. We believe that substantially tighter limits of agreements are required for such novel respiratory rate technologies to be useful in the clinical environment and that depth-based measurements may provide the opportunity to accomplish this. The limits of agreement found for our study are indicative of a potentially more accurate RR monitoring technology which we believe is due to the ability to parse out large spatial regions of respiratory modulations, although further testing of depth-based methods in a clinical environment is required.

**Fig. 7:**
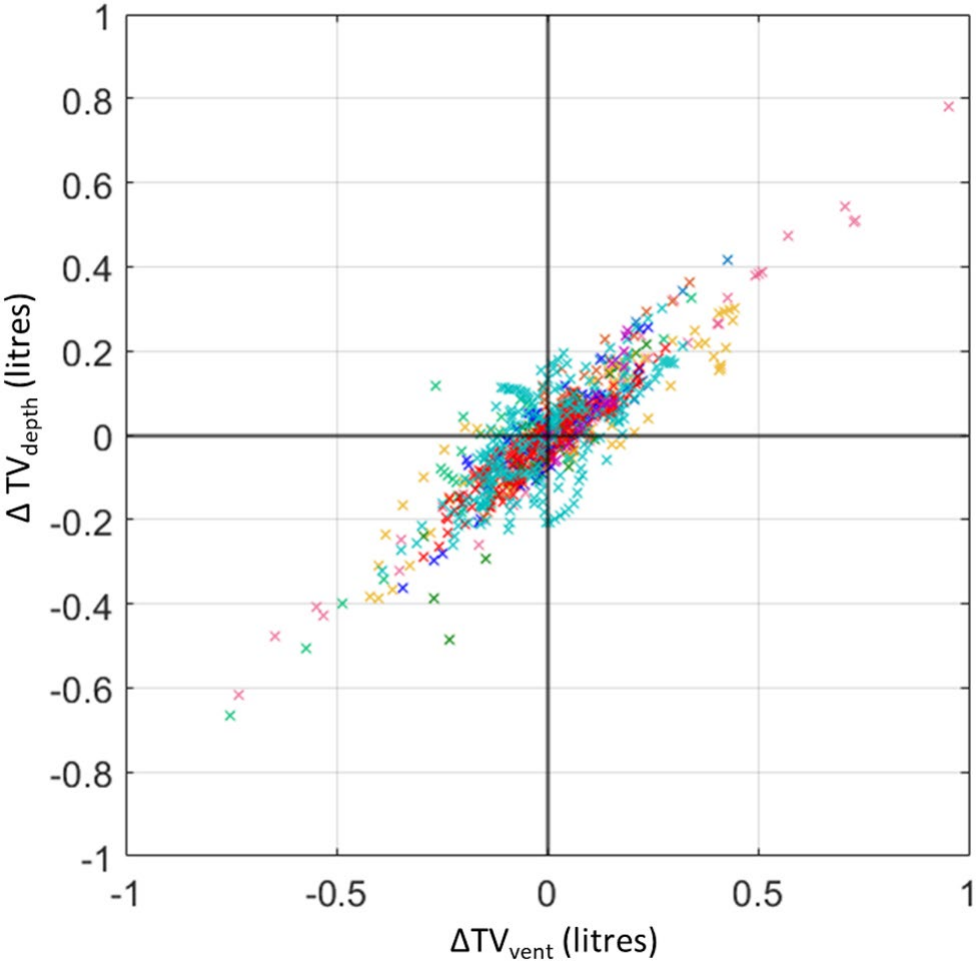
Concordance plot of tidal volume. A time delay of 30 s was used to construct the plot

The depth-based tidal volume exhibited a high degree of correlation (R = 0.92). However, the resulting line of best fit, although distinct in nature, deviated markedly from the line of unity. However, the strong linear relationship that was observed indicates the potential for TV_depth_ to be a reliable trend monitor for tidal volume. To further investigate this, we constructed a concordance plot where the difference between the two parameters over a running time window (ΔTV_depth_ and ΔTV_vent_) are plotted against each other [24]. We can see from the resulting concordance plot (Fig. 7) that the majority of data lie in the top right hand and bottom left hand quadrants, indicative of a strong trending behaviour. A concordance value can be computed by counting the number of data points in these two quadrants relative to the total data count. The concordance was found to be 0.88 for these tidal volume data indicating a high degree of trending between the two signals. It may be possible to construct a mapping from TV_depth_ to the actual tidal volume through a correction factor, however, achieving this in practice may be non-trivial due to need to account for various patient postures, the boundary morphology of the ROI, the presence of blankets, etc.

The study had several limitations. Motion was restricted as the participant was lying supine and remained relatively immobile during the acquisition hence a relatively simple algorithm was required. However, in practice, noise handling would also have to be incorporated to cope with patient motion—including change of posture or position in bed, hand and limb movements—and interference within the field of view of the camera – including clinical staff and equipment movement and patient hand motion across the region of interest. The development of a more robust algorithm for clinical practice would require the collection and analysis of large amounts of patient data acquired from across the spectrum of patient care. In addition, only a single subject was used. We had planned to extend the study to include more volunteers, however, due to the current global pandemic it was not possible to open it up to more subjects. The study had several strengths: off-the-shelf depth sensing camera technology was used requiring no hardware changes; the system is also simple to set up and operate, requiring no calibration; and, the use of a ventilator provided highly accurate reference values for respiration rate and tidal volume. In addition, we were able to capture respiratory rates over a relatively wide range of ‘normal’ breathing activity of 10 to 18 breaths per minute and included a wide range of tidal volumes from 0.1 to 1.5 L per breath.

We believe that this technology has major opportunities given the current drive for more remote and non-contact monitoring of the patient. This drive has been given particular impetus due to the current COVID-19 pandemic where technologies are being sought to minimize contact between the clinician and the patient. In addition to the monitoring of those pandemic disease states where respiratory function is a key indicator, we believe that the technology would be particularly useful in a range of other clinical settings, including: the post-anaesthesia care unit (PACU), where an early indication of respiratory depression through, for example, reductions in respiratory rate, tidal volume and/or opioid induced intermittent respiratory patterns would be useful; the neonatal intensive care unit (NICU), where there is a need to avoid excessive contact with the neonatal skin and where an indication of respiratory patterns, including apnea, could prove beneficial; for sleep monitoring, both in the sleep clinic and the home; for respiratory patients exhibiting a wide range of disease states, and in the general ward environment to provide a continuous measure of RR, which is a major vital sign.

The technology may be incorporated onto existing camera systems for observing the patient. In addition, further physiological and patient contextual information may be available from the same modality including patient activity monitoring, apnea identification, determination of presence in bed and fall detection. The technology has a number of key strengths including its ease of use and its ability to work through patient clothing and bed sheets, and it also operates with the lights turned off. These characteristics set it apart from physiological monitoring using other camera modalities which rely on the analysis of RGB image streams.

## Conclusion

The results demonstrate the potential for robust monitoring of respiratory rate and tidal volume trending using depth sensing camera equipment. Future work would aim to better understand the operating envelope of the technology through a series of benchtop and in-hospital tests involving a range of subjects with varying demographics and disease states to fully cover the operating range likely to be encountered in clinical practice. In addition, it is suggested that further investigation of the relationship between the depth-based respiratory volume measurement and the true tidal volume should be conducted.
